# Drug Pricing Evolution in Hepatitis C

**DOI:** 10.1371/journal.pone.0157098

**Published:** 2016-06-16

**Authors:** Nathalie Vernaz, François Girardin, Nicolas Goossens, Urs Brügger, Marco Riguzzi, Arnaud Perrier, Francesco Negro

**Affiliations:** 1 Medical Direction, Geneva University Hospitals, University of Geneva, Geneva, Switzerland; 2 Finance Direction, Geneva University Hospitals, Geneva, Switzerland; 3 Division of Clinical Pharmacology and Toxicology, Geneva University Hospitals, University of Geneva, Geneva, Switzerland; 4 Divisions of Gastroenterology and Hepatology, Geneva University Hospitals, University of Geneva, Geneva, Switzerland; 5 Winterthur Institute of Health Economics, Zurich University of Applied Sciences, Winterthur, Switzerland; 6 Division of General Internal Medicine, Geneva University Hospitals, University of Geneva, Geneva, Switzerland; 7 Divisions of Gastroenterology and Hepatology and of Clinical Pathology, Geneva University Hospitals, Geneva, Switzerland; Temple University School of Medicine, UNITED STATES

## Abstract

**Objective:**

We aimed to determine the association between the stepwise increase in the sustained viral response (SVR) and Swiss and United States (US) market prices of drug regimens for treatment-naive, genotype 1 chronic hepatitis C virus (HCV) infection in the last 25 years. We identified the following five steps in the development of HCV treatment regimens: 1) interferon (IFN)-α monotherapy in the early '90s, 2) IFN-α in combination with ribavirin (RBV), 3) pegylated (peg) IFN-α in combination with RBV, 4) the first direct acting antivirals (DAAs) (telaprevir and boceprevir) in combination with pegIFN-α and RBV, and 5) newer DAA-based regimens, such as sofosbuvir (which is or is not combined with ledipasvir) and fixed-dose combination of ritonavir-boosted paritaprevir and ombitasvir in combination with dasabuvir.

**Design:**

We performed a linear regression and mean cost analysis to test for an association between SVRs and HCV regimen prices. We conducted a sensitivity analysis using US prices at the time of US drug licensing. We selected randomized clinical trials of drugs approved for use in Switzerland from 1997 to July 2015 including treatment-naïve patients with HCV genotype 1 infection.

**Results:**

We identified a statistically significant positive relationship between the proportion of patients achieving SVRs and the costs of HCV regimens in Switzerland (with a bivariate ordinary least square regression yielding an R2 measure of 0.96) and the US (R2 = 0.95). The incremental cost per additional percentage of SVR was 597.14 USD in Switzerland and 1,063.81 USD in the US.

**Conclusion:**

The pricing of drugs for HCV regimens follows a value-based model, which has a stable ratio of costs per achieved SVR over 25 years. Health care systems are struggling with the high resource use of these new agents despite their obvious long-term advantages for the overall health of the population. Therefore, the pharmaceutical industry, health care payers and other stakeholders are challenged with finding new drug pricing schemes to treat the entire population infected with HCV.

## Introduction

The development and market approval of novel direct-acting antivirals (DAAs) have dramatically changed the hepatitis C virus (HCV) treatment landscape. For the first time, chronic HCV infection, a major human pathogen responsible for cirrhosis and hepatocellular carcinoma and a leading indication for liver transplantation, may be eradicated in all patients using an all-oral, short-duration, well-tolerated, safe, and highly efficacious treatment.[1]

A new nosological entity first identified in the 1970s, the so-called non-A, non-B hepatitis, was discovered as HCV in 1989.[2, 3] The first treatment consisted of recombinant interferon (IFN)-α monotherapy and was characterized by a low cure percentage (<10%) and severe side effects.[3] In subsequent decades, new drugs gradually increased the percentage of cure in a stepwise fashion.[2, 3] The IFN-α in combination with ribavirin (RBV) resulted in 34–42% cure rates. The substitution of standard IFN-α with its pegylated (peg) form, administered once per week, resulted in an increase in cure rates from 45% to 80% depending on the HCV genotype.[4, 5] The first DAAs were telaprevir (TVR) and boceprevir (BOC), which increased the cure rate up to 70–80% for HCV genotype 1.[6–8] Finally, the arrival of the new DAAs, such as sofosbuvir (SOF) by itself or in combination with ledipasvir (LDV), and the triple fixed-dose combination of ritonavir-boosted paritaprevir, ombitasvir (PTV/r/OBV) in combination with dasabuvir (DSV) led to unprecedented ~100% cure rates in several patient subgroups.[9–11] At the same time, two additional DAAs, simeprevir (SMV) and daclatasvir (DCV), were added to the already available armamentarium.[12–15] However, the new drug regimens were marketed at very high prices for each treatment compared to their predecessors, increasing the financial challenge for health care systems aiming to provide the entire HCV-infected population with access to those medicines. The cost of a 12-week SOF-based regimen in the United Sates (US) that contains pegIFN-α and RBV exceeded 90,000 USD at registration, which is much higher than the standard cost of 20,000 USD for historical IFN-α monotherapy.[16, 17]

Therefore, a better understanding of the determinants of HCV drug pricing is essential.[18] Key elements of drug pricing include the amount of money invested in research and development (R&D), production costs, efficacy, safety, ease of administration, duration of treatment, features of treatment comparators, innovation, international benchmarking, market size and market value.[19–22] Thus, we aimed to determine the potential association between the stepwise increase in the sustained viral response (SVR) and treatment regimen prices. We assessed the prices of different regimens at their market entry and the respective SVR rates, which are listed in the official Swiss label (available at http://www.swissmedicinfo.ch) for treatment-naive, genotype 1 chronic hepatitis C patients, corresponding to the most prevalent patient subgroup in our country.[1, 23] We also conducted a sensitivity analysis using US prices at the time of drug licensing.

## Materials and Methods

### Therapy stepping stones

We classified the different treatment regimens into five steps, as suggested by the literature (Table 1).[3, 24, 25]

**Table 1:** Approved drugs for HCV infection in Switzerland and the United States.

Steps	Regimen	Drugs	In combination with Interferon	Swissmedic approval	FDA approval
**Step 1**	Interferon-α monotherapy	IFN-α-2a	**-**	1997	
		IFN-α-2b	**-**	1998	
**Step 2**	Interferon-α associated with ribavirin	RBV	Yes	2002	2001
**Step 3**	Pegylated interferon-α associated with ribavirin	pegIFN-α-2a			2001
		pegIFN-α-2b	-	2003	2002
**Step 4**	First DAAs (serine protease inhibitors) associated with pegylated interferon-α and ribavirin	TVR, BOC	Yes	2011	2011
		SMV	Yes	2015	2013
**Step 5**	Second wave DAAs: nucleotidic and non-nucleosidic polymerase inhibitors, NS5A inhibitors, more serine protease inhibitors that are or are not associated with pegylated interferon-α and ribavirin	SOF	Yes	2014	2013
		SOF/LDV	No	2015	2014
		PTV/r/OBV +DSV	No	2014	2015

IFN: interferon; RBV: ribavirin; pegIFN: pegylated interferon; TVR telaprevir; BOC: boceprevir; SMV: simeprevir; SOF: sofosbuvir; LDV: ledipasvir; PTV/r/OBV: fixed dose combination of ritonavir-boosted paritaprevir and ombitasvir; DSV: dasabuvir

We selected all phase 3 randomized clinical trials (RCT) that enrolled previously untreated patients with chronic HCV genotype 1 infection and tested drugs that were approved by Swissmedic, which were therefore marketed in Switzerland from January 1^st^ 1997 to July 31^st^ 2015. For each of these drugs regimens, we used the SVR rate as reported in the studies at the time of the Swissmedic marketing authorization request (http://www.swissmedic.org); Swissmedic is the Swiss agency that authorizes and supervises drugs. The drug price, dosage and duration were based on the recommended Swissmedic guidelines for HCV treatment-naive patients with genotype 1 infection during the study period. For older drug regimens that are not listed in the Swissmedic database, we used data from the corresponding US agency (Food and Drug Administration, FDA). Finally, because data for IFN-α were not recorded in either database, we contacted the pharmaceutical companies to determine which published studies contributed to drug marketing. [4,5] SVR was defined as undetectable HCV RNA in the serum 12 weeks after the end of treatment for DAA-based regimens and 24 weeks for IFN-α based regimens.[26]

### Swiss market access

In Switzerland, market access is conditional on a two-step decision process. First, a drug must be approved by Swissmedic.[27] Then, the Federal Office of Public Health (FOPH) decides whether or not to include the medication in the list of drugs reimbursed by the mandatory health insurance scheme based on a recommendation by the Federal Drug Commission (FDC). The FDC evaluates the value of new drugs according to the following three criteria: effectiveness, appropriateness, and efficiency. Furthermore, the FDC applies reference pricing using a basket of nine European countries. Finally, the FDC considers the budget impact by comparing the total costs of the new treatment regimen with the standard of care. The FOPH then negotiates the final price with the manufacturer and re-evaluates the drug price every three years. We performed analysis from a third-party payer perspective.

### Cost calculation

The market prices of IFN-α, pegIFN-α and RBV have changed throughout the past 25 years. Therefore, we controlled for inflation by using the market price of IFN-α for step 1 in 1997, which was adjusted by the inflation rate for both Swiss and US pricing using an online calculator (fxtop.com/en/inflation-calculator.php). Likewise, for steps 2 and 3, we used the January 2003 prices of IFN-α, pegIFN-α and RBV, which were adjusted by the inflation rate. For the first and second wave DAAs included in steps 4 and 5, we used the launching market price and current price for pegIFN-α and RBV without adjusting for inflation. For the comparison of prices adjusted to the 2015 level, we considered an exchange rate of one CHF equal to one USD. Although RBV administration is usually weight-adapted, we arbitrarily considered a standard daily dose of 1,000 mg for all recipients. We assumed that the US drug costs were equal to the wholesale acquisition costs (WAC) as listed in the Red Book Online (http://www.redbook.com/redbook/online). For regimens requiring RBV, and when many manufacturers shared the RBV market, we selected the cheapest regimen. We also calculated the ratio of costs per SVR (costs/SVR) for each regimen, representing the costs to cure one patient.

### Statistical analysis

We used two methods to assess the HCV therapy pricing model. First, we plotted the costs per treatment of the twenty-two regimens against the rate of SVR for both Switzerland and the US. It is noteworthy that ordering according to the SVR rate corresponds to the stepwise increase in the cure over time described in the literature.[2, 3] Because the scatter diagram indicates a linear relationship, we tested for the linear correlation between the two variables by the standard Pearson correlation coefficient and R^2^ of a bivariate linear regression.[28] Both represent the overall fit of a linear model. Second, we measured the mean and standard deviation of the costs and costs per SVR for the five HCV treatment steps.[3, 24] We used Eviews 8 software (QMS) for the statistical analysis.

## Results

Twenty-two RCTs were included in our study, comprising a total of 5,900 patients (Table 2).

**Table 2:** Treatments, duration, costs and costs per SVR of HCV treatments over time in Switzerland and the US (Table A in S1 File).

Treatment	Study	Treatment duration, weeks	Patients, n SVR achieved/n total	SVR, %	Swiss Costs USD	Costs per SVR in Switzerland USD	US costs USD	US costs per SVR in US USD
**Step 1**								
IFN-α-2a	FDA Roferon label[29]	48	20/173	11.56%	9,544	82,561	6,148	53,183
IFN-α-2b	Reference[4]	48	37/303	12.21%	9,706	79,492	6,046	49,517
**Step 2**								
IFN-α-2b+RBV	CARRY FORWARD[30]	48	111/328	33.84%	23,799	70,328	25,543	75,482
IFN-α-2a+RBV	NV15801[31]	48	100/285	35.09%	20,951	59,706	24,584	70,060
**Step 3**								
pegIFN-α-2a+RBV	ADVANCE[6]	48	158/361	43.77%	34,575	78,992	37,222	85,040
pegIFN-α-2a+RBV	NV15801[31]	48	132/298	44.30%	34,575	78,047	37,222	84,023
pegIFN-α-2b+RBV	CARRY FORWARD[30]	48	58/122	47.54%	33,637	70,755	32,979	69,371
pegIFN-α-2a+RBV	QUEST-1[13]	48	65/130	50.00%	34,575	69,150	37,222	74,444
pegIFN-α-2a+RBV	QUEST-2[14]	48	67/134	50.00%	34,575	69,150	37,222	74,444
pegIFN-α-2a+RBV	NV15942[31]	48	142/271	52.40%	34,575	65,983	37,222	71,034
**Step 4**								
BOC+ pegIFN-α-2a+RBV	SPRINT-2[7]	4+44 (73%)^*^	233/368	63.32%	42,566	67,224	57,741	91,189
TVR+ pegIFN-α-2a+RBV	OPTIMIZE[32]	12+36 (67%)^†^	270/371	72.78%	47,304	64,996	75,210	103,339
TVR+ pegIFN-α-2a+RBV	OPTMIZE[32]	12+36 (69%)^†^	274/369	74.25%	47,091	63,422	74,818	100,765
TVR+ pegIFN-α-2a+RBV	ADVANCE[6]	12+36 (68%)^†^	285/363	78.51%	47,240	60,171	75,092	95,646
SMV+ pegIFN-α+RBV	QUEST-1[13]	12+12 (85%)^¶^	210/264	79.55%	44,833	56,358	85,891	107,971
SMV+ pegIFN-α+RBV	QUEST-2[14]	12+12 (91%)^¶^	209/257	81.32%	44,512	54,737	87,741	107,896
**Step 5**								
SOF+ pegIFN-α-2a+RBV	NEUTRINO[11]	12	262/292	89.73%	62,955	70,160	93,808	104,545
SOF/LDV	ION-3[10]	12	208/216	96.30%	62,363	64,759	94,500	98,131
PTV/r/OBV+DSV+ RBV	SAPHIRE-I[12]	12	455/473	96.19%	63,946	66,479	86,215	89,630
PTV/r/OBV+DSV+ RBV	PEARL-IV[9]	12	97/100	97.00%	63,946	65,924	86,215	88,881
SOF/LDV	ION-1[33]	12	210/213	98.59%	62,363	63,255	94,500	95,852
PTV/r/OBV+DSV	PEARL-III[9]	12	209/209	100%	61,956	61,956	83,319	83,319

* The stopping rule for response-guided therapy BOC included patients with undetectable HCV RNA from treatment weeks 8 to 24

^†^ A patient qualified for a shortened TVR therapy duration for HCV RNA <25 IU/ml at weeks 4 and 12.

^¶^ SMV response-guided therapy involved stopping the treatment after 24 weeks for patients with HCV RNA <25 IU/ml at week 4 (undetectable or detectable) and <25 IU/ml at week 12 (undetectable)

Two clinical trials were included for the IFN-α monotherapy step (n = 476 patients)[4, 29], two studies for the association between IFN-α and RBV (n = 613 patients)[5, 31], six for step 3 between pegIFN-α and RBV (n = 1,316 patients)[5, 6, 13, 14, 31], six for the first DAAs (n = 1,992 patients)[6, 7, 13, 14, 32], and six for the newer DAAs (n = 1,503 patients).[9–12, 33]

Table 2 provides the costs and costs per SVR for each individual treatment regimen and shows a steady cost increase paralleling the increase in the SVR rate for each treatment step. From step 4 onward, the treatment durations were shortened to 24 or even 12 weeks, and pegIFN-α could be omitted for most regimens at step 5.

**Fig 1:**
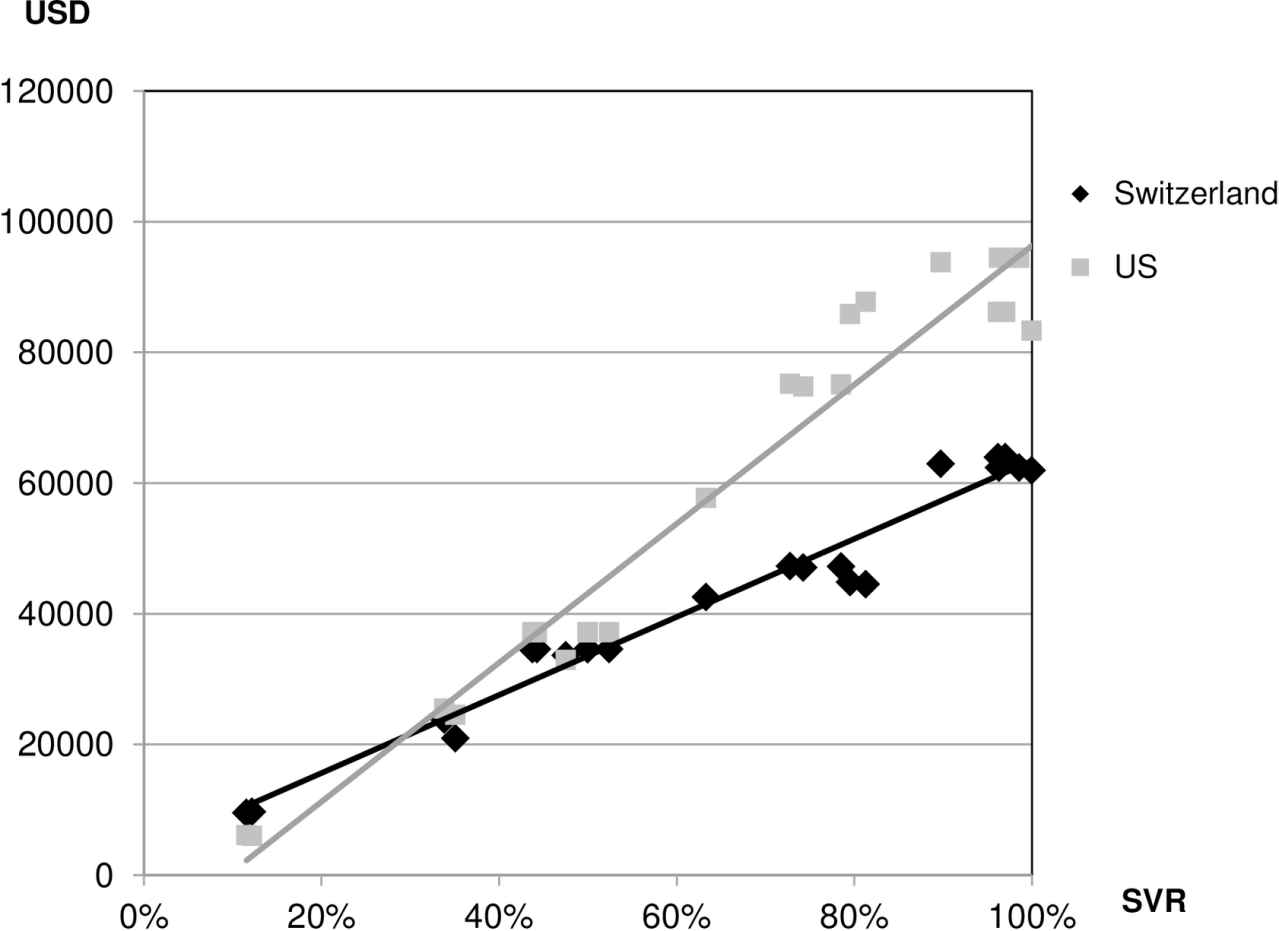
Scatter plot of costs against SVR of HCV therapies in Switzerland (ρ = 0.98, R^2^ = 0.96) and the US (ρ = 0.98, R^2^ = 0.95).

The scatter plot in Fig 1 suggests a positive linear association between SVR and the cost of HCV treatment regimens using Swiss cost figures. A high degree of positive linear dependence is indicated by a Pearson correlation coefficient that is close to unity (ρ = 0.98 for Switzerland and ρ = 0.98 for the US), corresponding to an R^2^ of 0.96 for Switzerland and 0.95 for the US. (Note that the two statistical measures are related by the square-root function.) The incremental costs per additional percentage point of SVR were estimated as 597.14 USD in Switzerland and 1,063.81 USD in the US by the slope coefficient of the respective regression model (Tables B-E in S1 File).

Table 3 details the mean costs and costs per SVR for each new HCV treatment step.

**Table 3:** Mean SVR, mean costs, costs per SVR and confidence intervals (CI) of HCV treatments over time in Switzerland and the US (Tables F-J in S1 File).

Treatment regimens	Mean SVR, % (95% CI)	Mean costs, in Switzerland, USD (95% CI)	Mean costs per SVR in Switzerland, USD (95% CI)	Mean costs in US, USD (95% CI)	Mean costs per SVR in US USD (95% CI)
**Step 1:** IFN-α monotherapy	11.89% (5.24%-18.53%)	9,625 (7,736–11,514)	81,026 (74,128–87,924)	6,097 (-3,580–15,771)	51,350 (41,406–61,295)
**Step 2:** IFN-α and RBV	34.47% (27.82%-41.11%)	22,375 (20,486–24,264)	65,017 (58,119–71,915)	25,064 (15,869–35,217)	72,771 (64,826–82,715)
**Step 3:** pegIFN-α and RBV	48.00% (44.16%-51.84%)	34,419 (33,328–35,509)	72,013 (68,030–75,996)	36,515 (30,928–42,102)	76,393 (70,651–82,134)
**Step 4**: First DAA protease inhibitors*, pegIFN-α and RBV	74.96% (71.12%-78.79%)	45,591 (44,500–46,682)	61,151 (57169–65,134)	76,082 (70-495-81,669)	101,134 (95,393–106,876)
**Step 5**: New DAA^†^ ± pegIFN-α and RBV	96.30% (92.46%-1.00%)	62,922 (61,831–64,012)	65,422 (61,440–69,405)	89,760 (84,172–95,347)	93,393 (87,651–99,134)

CI: Confidence Interval

* boceprevir; telaprevir; simeprevir

^†^ sofosbuvir; ledipasvir; ritonavir-boosted paritaprevir, ombitasvir, and dasabuvir

**Fig 2:**
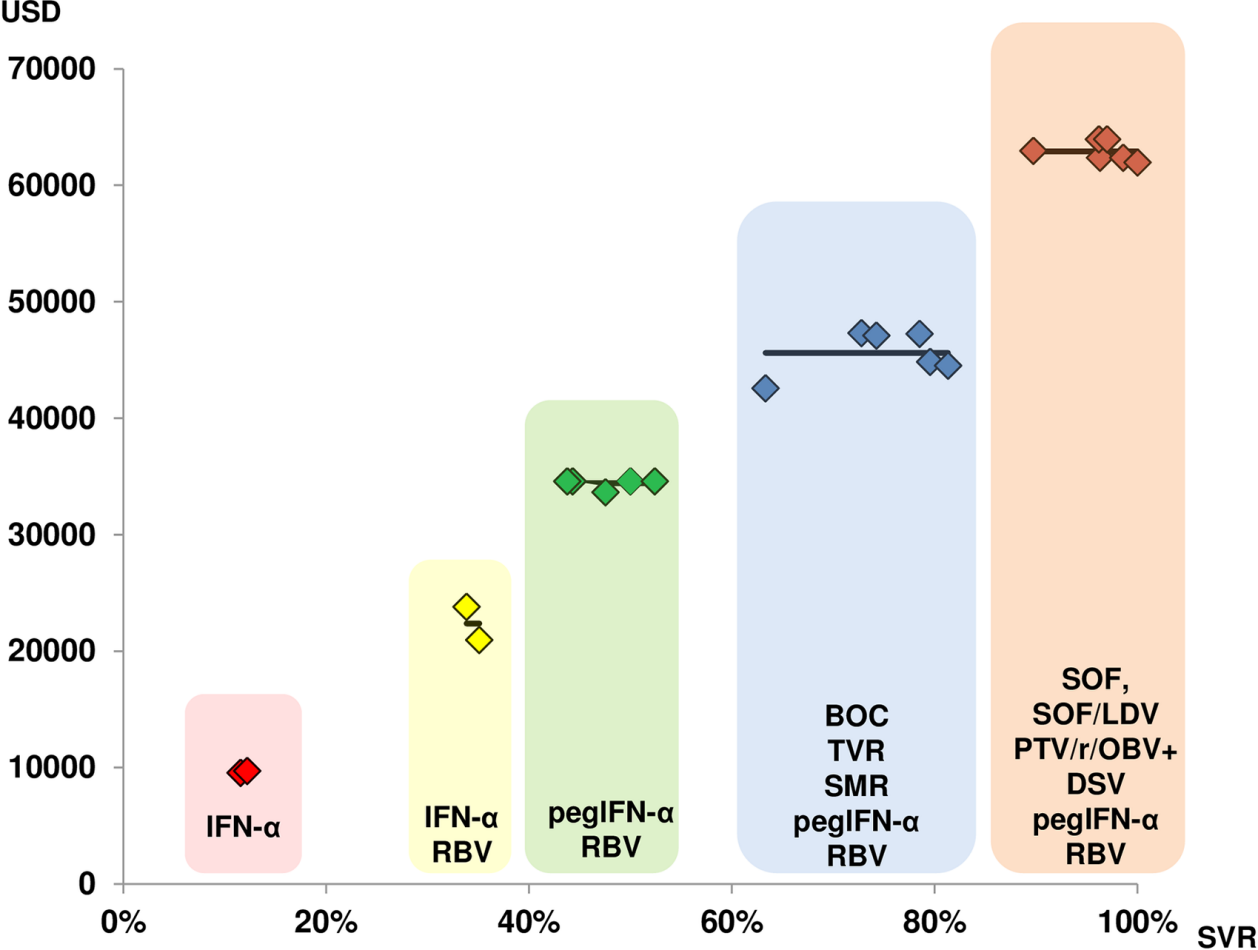
Costs associated with the five steps in HCV therapy development over time (SVR steadily increases with time).

**Fig 3:**
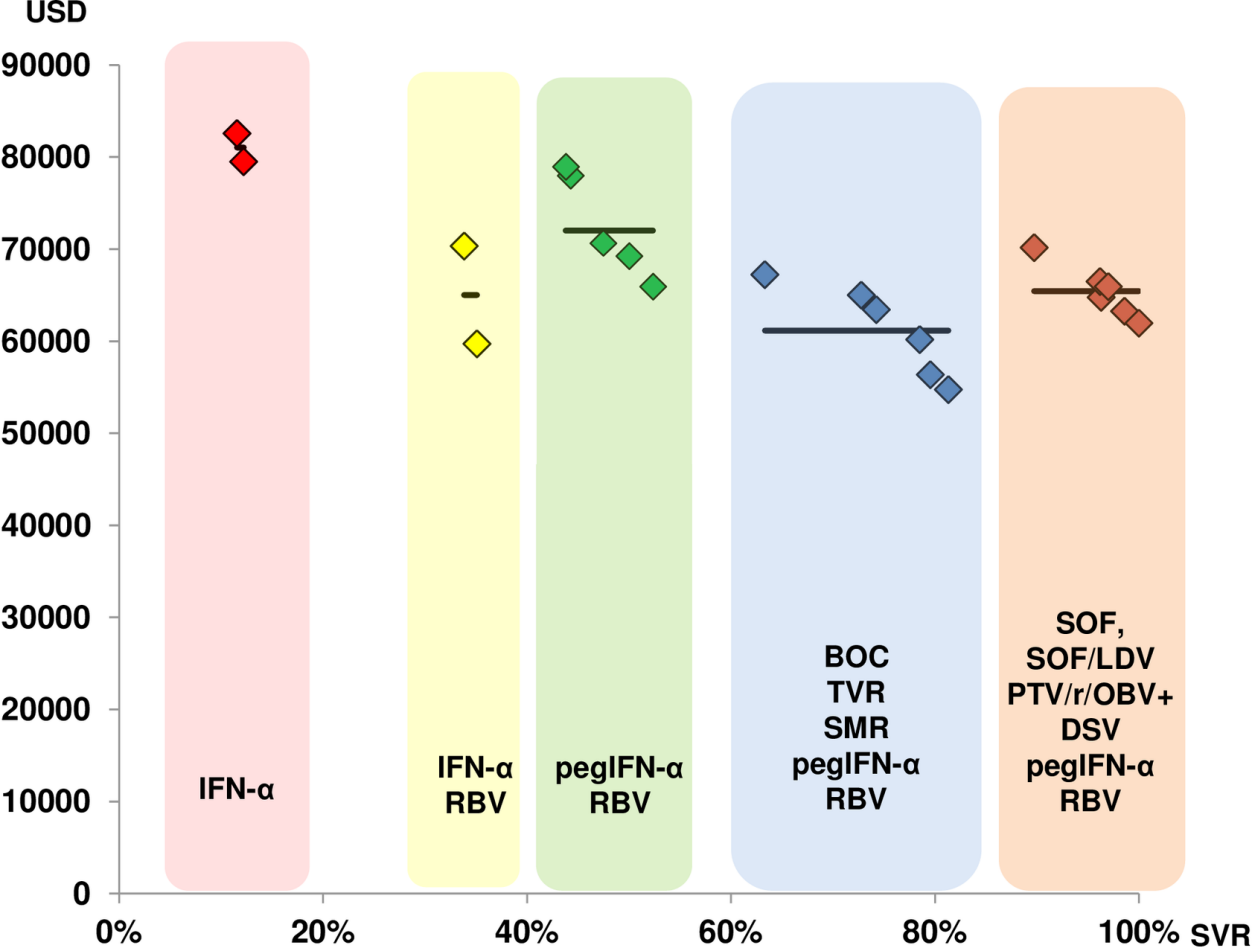
Cost per SVR associated with SVR rates corresponding to the five steps in the development of HCV therapies over time (SVR steadily increases with time).

These results confirmed the close association between the mean costs per treatment step and the mean SVR rate. Fig 2 shows both the individual and the mean costs for each treatment step, according to the increasing SVR in Switzerland. Finally, Fig 3 shows that the costs per achieved HCV cure, expressed as the SVR, are relatively stable.

## Discussion

Our results show that the costs of drug regimens for treating HCV increased steadily over time, both in Switzerland and in the US, according to the stepwise approval of new antiviral drugs, and in close correlation with the corresponding HCV cure rates. This resulted in relatively stable costs to cure a treatment-naive patient with HCV genotype 1 infection, irrespective of the fact that drug costs were higher in the US than in Switzerland. Our results are consistent with a value-based pricing model in which drug costs are closely related to their clinical efficacy. The relatively stable ratio of costs per achieved SVR, with the exception of the first treatment step, suggests that the societal willingness to pay for a specific health gain (i.e., one SVR) have remained relatively stable over time, at least in Switzerland.

For the HCV market, pharmaceutical companies price their drugs based on the value of SVR and profit maximization rather than the costs of R&D, production, marketing and distribution or the volume of drugs used.[19, 34, 35] These were also the conclusions of a recent bipartisan US Congress investigation on the SOF drug pricing strategy.[36] Newer antiviral drugs for HCV infection have significantly benefited from an improved understanding of viral biology and drug targets in the field of HIV infection.[2] Moreover, and in contrast to biologic agents in the fields of cancer and immunology, HCV drug production costs are very low.[37]

Drug prices evolved in line with the cure rate from a mere 10% to almost 100% over a 25-year period. Therefore, we could argue that the “very high” nominal prices of the recently introduced HCV DAAs are justifiable. On the other hand, DAAs have other significant advantages, such as improved safety and tolerability, shorter treatment durations, and entirely oral administration. These strong advantages–not present at the time of poorly tolerated IFN-α-based regimens–were not factored into the price of the first DAAs or into the price of the DAAs for the newer wave. Additionally, the purported innovation conferred by the introduction of long-awaited, IFN-free regimens was not responsible for boosting the market price.[38]

Some antiviral therapies have not only unprecedented high cure rates but are also safe and well tolerated, which makes them theoretically useful for treating nearly the entire infected population, at variance with the IFN-based therapies used in the early 1990s, which were associated with many safety issues and therefore of relatively limited use.[24] Also, antiviral therapies potentially prevent the dramatic and costly long-term complications of HCV infection, such as cirrhosis and hepatocellular carcinoma, loss of productivity and reduced quality of life.[23, 17] However, nominal drug costs fall short of emphasizing the true financial burden of large-scale treatment, despite their cost-effectiveness. [39] A number of analyses on novel HCV treatments have been published, demonstrating that expensive drugs can still be cost-effective.[1, 16, 40–43] In a recent analysis based on US costs, the incremental cost effectiveness ratios of DAAs compared to a reference treatment strategy, based on BOC, RBV and pegIFN-α, varied between 14,432 and 70,097 USD per additional QALY for genotype 1 infection, and they were highly sensitive to nominal drug prices.[41] For instance, a combination of SOF/LVD could be cost-saving as long as the weekly cost of SOF was reduced from 7,000 USD to less than 5,500 USD. Nevertheless, such projections take into account the long-term savings of curing patients of HCV, while the weight of treatment costs on health systems is immediate. Additionally, a drug that is considered cost-effective for an individual patient may still be unaffordable for the health care system, which depends on the disease prevalence in the general population.[44, 45] Thus, the cost-effectiveness of a drug that is measured at the patient level does not correspond to the cost-affordability of the drug at the population level.[39, 43–45] For this reason, varying degrees of restrictions have been introduced. In Switzerland, only patients with advanced fibrosis (Metavir F3), compensated cirrhosis (Metavir F4) or who are awaiting liver transplantation initially had access to reimbursement for DAAs by the mandatory health insurance at the time of their first approval. To address this situation, European countries implemented different policies.[46] One of them is price negotiation as more than one pharmaceutical company enters the hepatitis C market.[43] From August 2015, the Swiss FOPH extended the use of DAAs to patients with fibrosis stage F2, a lower stage of fibrosis. This extension was accompanied by a market price reduction. The Tuscany region (Italy) has proposed a more sophisticated tool, i.e., a tendering scheme that extends the accessibility to DAA for approximately 20,000 patients who have milder disease.[47] As a further example, in the field of other diseases, Novartis recently declared the launch of their new heart failure drug, Entresto^®^ (sacubitril associated to valsartan), offering a high rebate while subsequently increasing prices if the new drug reduces hospitals visits.[48]

Our study had limitations. First, we included only treatment-naive patients infected with HCV genotype 1. However, this is the largest subgroup of hepatitis C patients in Switzerland, and it is sufficiently representative for studying the pricing business model.[25] Moreover, HCV genotype 1 has been the most difficult genotype to treat for the last two decades, justifying the efforts to develop novel genotype-specific drugs. Second, prices are negotiated over time, and the market price at the time of licensing may not necessarily reflect the future, evolving situation.

In conclusion, there is a relatively stable ratio of costs per cured patient over time, resulting in a strong positive correlation between the HCV cure rate and costs per treatment. This is an indication that pharmaceutical companies used a value-based pricing model for HCV treatments. The finding is in line with a claim by Ezekiel J. Emanuel, who stated that high HCV prices are not fully accounted by high costs of R&D or risks associated with drug development. [49] Health care systems, even of wealthy countries, such as Switzerland and the US, are struggling with the high budget impact of these new agents. Ironically, the issue is caused by the very high effectiveness of the DAAs and the willingness to pay for a specific health gain (i.e. one SVR) set 25 years ago, with the consequence of high prices due to the high patient value. Nevertheless, the current pricing of antiviral drugs against HCV does not allow treatment of all HCV-infected patients despite the obvious long-term advantages in terms of population health. Therefore, the pharmaceutical industry, health care payers and stakeholders are challenged with finding new pricing schemes to treat the entire population for new drugs that are highly effective when the disease prevalence is high.

## Supporting Information

S1 File Table A in S1 File: Data set: SVR, Swiss costs (USD), United States (US) costs (USD), cost per SVR in Switzerland (USD), costs per SVR in US (USD), stepping stones dummy variables. Table B in S1 File: The Swiss incremental costs per additional percentage point of SVR regression coefficients. Table C in S1 File: The Swiss incremental costs per additional percentage point of SVR regression table output. Table D in S1 File: The US incremental costs per additional percentage point of SVR regression coefficients. Table E in S1 File: The US incremental costs per additional percentage point of SVR table output. Table F in S1 File: Mean SVR, 95% CI output. Table G in S1 File: Mean costs and 95% CI in Switzerland output. Table H in S1 File: Mean costs per SVR and 95% CI in Switzerland output. Table I in S1 File: Mean costs and 95% CI in the US output. Table J in S1 File: Mean costs per SVR and 95% CI in the US output.
(DOCX)
