# Supplementary material for: Drug Pricing Evolution in Hepatitis C
Source: PLoS One. 2016 Jun 16;11(6):e0157098. doi: 10.1371/journal.pone.0157098 (PMC4911078; doi:10.1371/journal.pone.0157098)
Supplement: S1 File — Table A in S1 File: Data set: SVR, Swiss costs (USD), United States (US) costs (USD), cost per SVR in Switzerland (USD), costs per SVR in US (USD), stepping stones dummy variables. Table B in S1 File: The Swiss incremental costs per additional percentage point of SVR regression coefficients. Table C in S1 File: The Swiss incremental costs per additional percentage point of SVR regression table output. Table D in S1 File: The US incremental costs per additional percentage point of SVR regression coefficients. Table E in S1 File: The US incremental costs per additional percentage point of SVR table output. Table F in S1 File: Mean SVR, 95% CI output. Table G in S1 File: Mean costs and 95% CI in Switzerland output. Table H in S1 File: Mean costs per SVR and 95% CI in Switzerland output. Table I in S1 File: Mean costs and 95% CI in the US output. Table J in S1 File: Mean costs per SVR and 95% CI in the US output. (DOCX) [file pone.0157098.s001.docx]

**Supplementary Materials for: Drug pricing evolution in hepatitis C**

Nathalie Vernaz^1,2^*, François Girardin^1,3^, Nicolas Goossens^4^, Urs Brügger^5^, Marco Riguzzi ^5^, Arnaud Perrier^1, 6^, Francesco Negro^7^

^1^Medical Direction, ^2^Finance Direction, ^3^Division of clinical Pharmacology and Toxicology, ^4^Divisions of Gastroenterology and Hepatology, ^5^Winterthur Institute of Health Economics, ^6^Division of General internal Medicine, ^7^Divisions of Gastroenterology and Hepatology and of Clinical pathology, Geneva University Hospitals, University of Geneva, Zurich University of Applied Sciences, Switzerland

∗To whom correspondence should be addressed; E-mail: nathalie.vernaz@hcuge.ch

**Table A: Data set: SVR, Swiss costs (USD), United States (US) costs (USD), cost per SVR in Switzerland (USD), costs per SVR in US (USD), stepping stones dummy variables.**

|  |  | Swistzerland | | US | | Stepping stones dummy variables | | | | |
| --- | --- | --- | --- | --- | --- | --- | --- | --- | --- | --- |
|  | SVR | Swiss costs USD | Costs per SVR in Swizerland USD | US costs  USD | US costs per SVR in US USD | STEP1 | STEP1 | STEP3 | STEP4 | STEP5 |
| 1 | 0.115600 | 9544.000 | 82560.55 | 6148.000 | 53183.39 | 1.000000 | 0.000000 | 0.000000 | 0.000000 | 0.000000 |
| 2 | 0.122100 | 9706.000 | 79492.22 | 6046.000 | 49516.79 | 1.000000 | 0.000000 | 0.000000 | 0.000000 | 0.000000 |
| 3 | 0.338400 | 23799.00 | 70328.01 | 25543.00 | 75481.68 | 0.000000 | 1.000000 | 0.000000 | 0.000000 | 0.000000 |
| 4 | 0.350900 | 20951.00 | 59706.47 | 24584.00 | 70059.85 | 0.000000 | 1.000000 | 0.000000 | 0.000000 | 0.000000 |
| 5 | 0.443000 | 34575.00 | 78047.40 | 37222.00 | 84022.57 | 0.000000 | 0.000000 | 1.000000 | 0.000000 | 0.000000 |
| 6 | 0.437700 | 34575.00 | 78992.46 | 37222.00 | 85039.98 | 0.000000 | 0.000000 | 1.000000 | 0.000000 | 0.000000 |
| 7 | 0.475400 | 33637.00 | 70755.15 | 32979.00 | 69371.06 | 0.000000 | 0.000000 | 1.000000 | 0.000000 | 0.000000 |
| 8 | 0.500000 | 34575.00 | 69150.00 | 37222.00 | 74444.00 | 0.000000 | 0.000000 | 1.000000 | 0.000000 | 0.000000 |
| 9 | 0.500000 | 34575.00 | 69150.00 | 37222.00 | 74444.00 | 0.000000 | 0.000000 | 1.000000 | 0.000000 | 0.000000 |
| 10 | 0.524000 | 34575.00 | 65982.82 | 37222.00 | 71034.35 | 0.000000 | 0.000000 | 1.000000 | 0.000000 | 0.000000 |
| 11 | 0.633200 | 42566.00 | 67223.63 | 57741.00 | 91189.20 | 0.000000 | 0.000000 | 0.000000 | 1.000000 | 0.000000 |
| 12 | 0.727800 | 47304.00 | 64995.88 | 75210.00 | 103338.8 | 0.000000 | 0.000000 | 0.000000 | 1.000000 | 0.000000 |
| 13 | 0.742500 | 47091.00 | 63422.22 | 74818.00 | 100765.0 | 0.000000 | 0.000000 | 0.000000 | 1.000000 | 0.000000 |
| 14 | 0.785100 | 47240.00 | 60170.68 | 75092.00 | 95646.41 | 0.000000 | 0.000000 | 0.000000 | 1.000000 | 0.000000 |
| 15 | 0.795500 | 44833.00 | 56358.27 | 85891.00 | 107971.1 | 0.000000 | 0.000000 | 0.000000 | 1.000000 | 0.000000 |
| 16 | 0.813200 | 44512.00 | 54736.84 | 87741.00 | 107896.0 | 0.000000 | 0.000000 | 0.000000 | 1.000000 | 0.000000 |
| 17 | 0.897300 | 62955.00 | 70160.48 | 93808.00 | 104544.7 | 0.000000 | 0.000000 | 0.000000 | 0.000000 | 1.000000 |
| 18 | 0.963000 | 62363.00 | 64759.09 | 94500.00 | 98130.84 | 0.000000 | 0.000000 | 0.000000 | 0.000000 | 1.000000 |
| 19 | 0.961900 | 63946.00 | 66478.84 | 86215.00 | 89629.90 | 0.000000 | 0.000000 | 0.000000 | 0.000000 | 1.000000 |
| 20 | 0.970000 | 63946.00 | 65923.71 | 86215.00 | 88881.44 | 0.000000 | 0.000000 | 0.000000 | 0.000000 | 1.000000 |
| 21 | 0.985900 | 62363.00 | 63254.89 | 94500.00 | 95851.51 | 0.000000 | 0.000000 | 0.000000 | 0.000000 | 1.000000 |
| 22 | 1.000000 | 61956.00 | 61956.00 | 83319.00 | 83319.00 | 0.000000 | 0.000000 | 0.000000 | 0.000000 | 1.000000 |

**Table B : The Swiss incremental costs per additional percentage point of SVR regression coefficients**

| Dependent Variable: COST | | |  |  |
| --- | --- | --- | --- | --- |
| Method: Least Squares | | |  |  |
| Date: 04/30/16 Time: 08:15 | | |  |  |
| Sample: 1 22 | |  |  |  |
| Included observations: 22 | | |  |  |
| HAC standard errors & covariance (Bartlett kernel, Newey-West fixed | | | | |
| bandwidth = 3.0000) | | |  |  |
|  |  |  |  |  |
|  |  |  |  |  |
| Variable | Coefficient | Std. Error | t-Statistic | Prob. |
|  |  |  |  |  |
|  |  |  |  |  |
| SVR2 | 59714.62 | 2399.310 | 24.88825 | 0.0000 |
| C | 3666.176 | 1460.510 | 2.510203 | 0.0208 |
|  |  |  |  |  |
|  |  |  |  |  |
| R-squared | 0.961985 | Mean dependent var | | 41890.32 |
| Adjusted R-squared | 0.960085 | S.D. dependent var | | 16821.24 |
| S.E. of regression | 3360.681 | Akaike info criterion | | 19.16418 |
| Sum squared resid | 2.26E+08 | Schwarz criterion | | 19.26337 |
| Log likelihood | -208.8060 | Hannan-Quinn criter. | | 19.18755 |
| F-statistic | 506.1149 | Durbin-Watson stat | | 1.440509 |
| Prob(F-statistic) | 0.000000 | Wald F-statistic | | 619.4248 |
| Prob(Wald F-statistic) | 0.000000 |  |  |  |
|  |  |  |  |  |
|  |  |  |  |  |

**Table C : The Swiss incremental costs per additional percentage point of SVR regression table output**

| obs | Actual | Fitted | Residual | Residual Plot |
| --- | --- | --- | --- | --- |
| 1 | 9544.00 | 10569.2 | -1025.19 | \| . * \| . \| |
| 2 | 9706.00 | 10957.3 | -1251.33 | \| . * \| . \| |
| 3 | 23799.0 | 23873.6 | -74.6032 | \| . * . \| |
| 4 | 20951.0 | 24620.0 | -3669.04 | \| *. \| . \| |
| 5 | 34575.0 | 30119.8 | 4455.25 | \| . \| . * \| |
| 6 | 34575.0 | 29803.3 | 4771.74 | \| . \| . * \| |
| 7 | 33637.0 | 32054.5 | 1582.49 | \| . \| * . \| |
| 8 | 34575.0 | 33523.5 | 1051.51 | \| . \| * . \| |
| 9 | 34575.0 | 33523.5 | 1051.51 | \| . \| * . \| |
| 10 | 34575.0 | 34956.6 | -381.637 | \| . *\| . \| |
| 11 | 42566.0 | 41477.5 | 1088.53 | \| . \| * . \| |
| 12 | 47304.0 | 47126.5 | 177.524 | \| . * . \| |
| 13 | 47091.0 | 48004.3 | -913.281 | \| . *\| . \| |
| 14 | 47240.0 | 50548.1 | -3308.12 | \| * \| . \| |
| 15 | 44833.0 | 51169.2 | -6336.16 | \| * . \| . \| |
| 16 | 44512.0 | 52226.1 | -7714.10 | \|* . \| . \| |
| 17 | 62955.0 | 57248.1 | 5706.90 | \| . \| . * \| |
| 18 | 62363.0 | 61171.4 | 1191.65 | \| . \| * . \| |
| 19 | 63946.0 | 61105.7 | 2840.33 | \| . \| *. \| |
| 20 | 63946.0 | 61589.4 | 2356.64 | \| . \| *. \| |
| 21 | 62363.0 | 62538.8 | -175.819 | \| . * . \| |
| 22 | 61956.0 | 63380.8 | -1424.80 | \| . * \| . \| |

**Table D: The US incremental costs per additional percentage point of SVR regression coefficients:**

| Dependent Variable: US_COSTS |  |  |
| --- | --- | --- |

| Method: Least Squares | | |  |  |
| --- | --- | --- | --- | --- |
| Date: 04/15/16 Time: 10:59 | | |  |  |
| Sample: 1 22 | |  |  |  |
| Included observations: 22 | | |  |  |
|  |  |  |  |  |
|  |  |  |  |  |
| Variable | Coefficient | Std. Error | t-Statistic | Prob. |
|  |  |  |  |  |
|  |  |  |  |  |
| C | -10075.02 | 3710.897 | -2.714983 | 0.0133 |
| SVR2 | 106381.0 | 5341.712 | 19.91515 | 0.0000 |
|  |  |  |  |  |
|  |  |  |  |  |
| R-squared | 0.951994 | Mean dependent var | | 58020.91 |
| Adjusted R-squared | 0.949594 | S.D. dependent var | | 30123.72 |
| S.E. of regression | 6763.191 | Akaike info criterion | | 20.56289 |
| Sum squared resid | 9.15E+08 | Schwarz criterion | | 20.66207 |
| Log likelihood | -224.1917 | Hannan-Quinn criter. | | 20.58625 |
| F-statistic | 396.6133 | Durbin-Watson stat | | 0.736278 |
| Prob(F-statistic) | 0.000000 |  |  |  |
|  |  |  |  |  |
|  |  |  |  |  |

**Table E: The US incremental costs per additional percentage point of SVR table output**

| obs | Actual | Fitted | Residual | Residual Plot |
| --- | --- | --- | --- | --- |
| 1 | 6148.00 | 2222.62 | 3925.38 | \| . \| * . \| |
| 2 | 6046.00 | 2914.10 | 3131.90 | \| . \| * . \| |
| 3 | 25543.0 | 25924.3 | -381.310 | \| . * . \| |
| 4 | 24584.0 | 27254.1 | -2670.07 | \| . * \| . \| |
| 5 | 37222.0 | 37051.8 | 170.237 | \| . * . \| |
| 6 | 37222.0 | 36487.9 | 734.057 | \| . \|* . \| |
| 7 | 32979.0 | 40498.5 | -7519.51 | \| *. \| . \| |
| 8 | 37222.0 | 43115.5 | -5893.48 | \| .* \| . \| |
| 9 | 37222.0 | 43115.5 | -5893.48 | \| .* \| . \| |
| 10 | 37222.0 | 45668.6 | -8446.62 | \| * . \| . \| |
| 11 | 57741.0 | 57285.4 | 455.570 | \| . * . \| |
| 12 | 75210.0 | 67349.1 | 7860.93 | \| . \| .* \| |
| 13 | 74818.0 | 68912.9 | 5905.13 | \| . \| *. \| |
| 14 | 75092.0 | 73444.7 | 1647.30 | \| . \| * . \| |
| 15 | 85891.0 | 74551.1 | 11339.9 | \| . \| . * \| |
| 16 | 87741.0 | 76434.0 | 11307.0 | \| . \| . * \| |
| 17 | 93808.0 | 85380.7 | 8427.35 | \| . \| . * \| |
| 18 | 94500.0 | 92369.9 | 2130.12 | \| . \| * . \| |
| 19 | 86215.0 | 92252.9 | -6037.87 | \| * \| . \| |
| 20 | 86215.0 | 93114.6 | -6899.55 | \| * \| . \| |
| 21 | 94500.0 | 94806.0 | -306.009 | \| . * . \| |
| 22 | 83319.0 | 96306.0 | -12987.0 | \|* . \| . \| |

**Mean SVR, mean costs, costs per SVR and confidence intervals (CI) of HCV treatments over time (Table 3)**

**Table F: Mean SVR, 95% CI output**

| Dependent Variable: SVR2 | | |  |  |  |  |  | Coefficient Confidence Intervals | | |  |  |
| --- | --- | --- | --- | --- | --- | --- | --- | --- | --- | --- | --- | --- |
| Method: Least Squares | | |  |  |  |  |  | Date: 04/30/16 Time: 07:57 | | |  |  |
| Date: 04/30/16 Time: 07:28 | | |  |  |  |  |  | Sample: 1 22 | |  |  |  |
| Sample: 1 22 |  | |  |  |  |  |  | Included observations: 22 | | |  |  |
| Included observations: 22 | | |  |  |  |  |  |  |  |  |  |  |
|  | |  |  |  |  |  |  |  |  |  | 95% CI |  |
| Variable | | Coefficient | Std. Error | t-Statistic | Prob. |  |  | Variable | Coefficient |  | Low | High |
|  | |  |  |  |  |  |  |  |  |  |  |  |
| STEP1 | | 0.11885 | 0.031512 | 3.771623 | 0.0015 |  |  | STEP1 | 0.11885 |  | 0.052366 | 0.185334 |
| STEP2 | | 0.34465 | 0.031512 | 10.93723 | 0 |  |  | STEP2 | 0.34465 |  | 0.278166 | 0.411134 |
| STEP3 | | 0.480017 | 0.018193 | 26.38433 | 0 |  |  | STEP3 | 0.480017 |  | 0.441632 | 0.518401 |
| STEP4 | | 0.74955 | 0.018193 | 41.19935 | 0 |  |  | STEP4 | 0.74955 |  | 0.711166 | 0.787934 |
| STEP5 | | 0.963017 | 0.018193 | 52.93264 | 0 |  |  | STEP5 | 0.963017 |  | 0.924632 | 1.001401 |
|  | |  |  |  |  |  |  |  |  |  |  |  |
| R-squared | | 0.978939 | Mean dependent var | | 0.640114 |  |  |  |  |  |  |  |
| Adjusted R-squared | | 0.973984 | S.D. dependent var | | 0.276288 |  |  |  |  |  |  |  |
| S.E. of regression | | 0.044564 | Akaike info criterion | | -3.187056 |  |  |  |  |  |  |  |
| Sum squared resid | | 0.033761 | Schwarz criterion | | -2.939092 |  |  |  |  |  |  |  |
| Log likelihood | | 40.05762 | Hannan-Quinn criter. | | -3.128644 |  |  |  |  |  |  |  |
| Durbin-Watson stat | | 1.879962 |  |  |  |  |  |  |  |  |  |  |

**Table G: Mean costs and 95% CI in Switzerland output**

| Dependent Variable: COST_CH | | |  |  |  |  | Coefficient Confidence Intervals | | |  |  |
| --- | --- | --- | --- | --- | --- | --- | --- | --- | --- | --- | --- |
| Method: Least Squares | |  |  |  |  |  | Date: 04/30/16 Time: 07:43 | |  |  |  |
| Date: 04/30/16 Time: 07:43 | |  |  |  |  |  | Sample: 1 22 |  |  |  |  |
| Sample: 1 22 |  |  |  |  |  |  | Included observations: 22 | |  |  |  |
| Included observations: 22 | |  |  |  |  |  |  |  |  |  |  |
|  |  |  |  |  |  |  |  |  |  | 95% CI |  |
| Variable | Coefficient | Std. Error | t-Statistic | Prob. |  |  | Variable | Coefficient |  | Low | High |
|  |  |  |  |  |  |  |  |  |  |  |  |
| STEP1 | 9625 | 895.2692 | 10.75096 | 0 |  |  | STEP1 | 9625 |  | 7736.147 | 11513.85 |
| STEP2 | 22375 | 895.2692 | 24.99248 | 0 |  |  | STEP2 | 22375 |  | 20486.15 | 24263.85 |
| STEP3 | 34418.67 | 516.8839 | 66.58878 | 0 |  |  | STEP3 | 34418.67 |  | 33328.14 | 35509.2 |
| STEP4 | 45591 | 516.8839 | 88.20356 | 0 |  |  | STEP4 | 45591 |  | 44500.47 | 46681.53 |
| STEP5 | 62921.5 | 516.8839 | 121.7324 | 0 |  |  | STEP5 | 62921.5 |  | 61830.97 | 64012.03 |
|  |  |  |  |  |  |  |  |  |  |  |  |
| R-squared | 0.995414 | Mean dependent var | | 41890.32 |  |  |  |  |  |  |  |
| Adjusted R-squared | 0.994335 | S.D. dependent var | | 16821.24 |  |  |  |  |  |  |  |
| S.E. of regression | 1266.102 | Akaike info criterion | | 17.32199 |  |  |  |  |  |  |  |
| Sum squared resid | 27251235 | Schwarz criterion | | 17.56995 |  |  |  |  |  |  |  |
| Log likelihood | -185.5419 | Hannan-Quinn criter. | | 17.3804 |  |  |  |  |  |  |  |
| Durbin-Watson stat | 2.183295 |  |  |  |  |  |  |  |  |  |  |

**Table H: Mean costs per SVR and 95% CI in Switzerland output**

| Dependent Variable: CHCOST_PER_SVR2 | | | | |  |  |  | Coefficient Confidence Intervals | | |  |  |
| --- | --- | --- | --- | --- | --- | --- | --- | --- | --- | --- | --- | --- |
| Method: Least Squares | |  | |  |  |  |  | Date: 04/30/16 Time: 07:54 | |  |  |  |
| Date: 04/30/16 Time: 07:53 | |  | |  |  |  |  | Sample: 1 22 |  |  |  |  |
| Sample: 1 22 |  |  | |  |  |  |  | Included observations: 22 | |  |  |  |
| Included observations: 22 | |  | |  |  |  |  |  |  |  |  |  |
|  |  |  | |  |  |  |  |  |  |  | 95% CI |  |
| Variable | Coefficient | Std. Error | | t-Statistic | Prob. |  |  | Variable | Coefficient |  | Low | High |
|  |  |  | |  |  |  |  |  |  |  |  |  |
| STEP1 | 81026.39 | 3269.482 | | 24.78264 | 0 |  |  | STEP1 | 81026.39 |  | 74128.38 | 87924.39 |
| STEP2 | 65017.24 | 3269.482 | | 19.8861 | 0 |  |  | STEP2 | 65017.24 |  | 58119.24 | 71915.25 |
| STEP3 | 72012.97 | 1887.636 | | 38.14981 | 0 |  |  | STEP3 | 72012.97 |  | 68030.41 | 75995.54 |
| STEP4 | 61151.25 | 1887.636 | | 32.39567 | 0 |  |  | STEP4 | 61151.25 |  | 57168.69 | 65133.82 |
| STEP5 | 65422.17 | 1887.636 | | 34.65825 | 0 |  |  | STEP5 | 65422.17 |  | 61439.6 | 69404.73 |
|  |  |  | |  |  |  |  |  |  |  |  |  |
| R-squared | 0.678811 | Mean dependent var | | | 67436.62 |  |  |  |  |  |  |  |
| Adjusted R-squared | 0.603237 | S.D. dependent var | | | 7340.543 |  |  |  |  |  |  |  |
| S.E. of regression | 4623.746 | Akaike info criterion | | | 19.91251 |  |  |  |  |  |  |  |
| Sum squared resid | 3.63E+08 | Schwarz criterion | | | 20.16048 |  |  |  |  |  |  |  |
| Log likelihood | -214.0377 | Hannan-Quinn criter. | | | 19.97093 |  |  |  |  |  |  |  |
| Durbin-Watson stat | 1.99875 |  |  | |  |  |  |  |  |  |  |  |

Table I: **Mean costs and 95% CI in the US output**

| Dependent Variable: US_COSTS | | |  |  |  |  | Coefficient Confidence Intervals | | |  |  |
| --- | --- | --- | --- | --- | --- | --- | --- | --- | --- | --- | --- |
| Method: Least Squares | |  |  |  |  |  | Date: 04/30/16 Time: 08:07 | |  |  |  |
| Date: 04/30/16 Time: 08:06 | |  |  |  |  |  | Sample: 1 22 |  |  |  |  |
| Sample: 1 22 |  |  |  |  |  |  | Included observations: 22 | |  |  |  |
| Included observations: 22 | |  |  |  |  |  |  |  |  |  |  |
|  |  |  |  |  |  |  |  |  |  | 95% CI |  |
| Variable | Coefficient | Std. Error | t-Statistic | Prob. |  |  | Variable | Coefficient |  | Low | High |
|  |  |  |  |  |  |  |  |  |  |  |  |
| STEP1 | 6097 | 4586.71 | 1.329275 | 0.2013 |  |  | STEP1 | 6097 |  | -3580.112 | 15774.11 |
| STEP2 | 25063.5 | 4586.71 | 5.464374 | 0 |  |  | STEP2 | 25063.5 |  | 15386.39 | 34740.61 |
| STEP3 | 36514.83 | 2648.138 | 13.78887 | 0 |  |  | STEP3 | 36514.83 |  | 30927.75 | 42101.92 |
| STEP4 | 76082.17 | 2648.138 | 28.73044 | 0 |  |  | STEP4 | 76082.17 |  | 70495.08 | 81669.25 |
| STEP5 | 89759.5 | 2648.138 | 33.89532 | 0 |  |  | STEP5 | 89759.5 |  | 84172.42 | 95346.58 |
|  |  |  |  |  |  |  |  |  |  |  |  |
| R-squared | 0.962464 | Mean dependent var | | 58020.91 |  |  |  |  |  |  |  |
| Adjusted R-squared | 0.953632 | S.D. dependent var | | 30123.72 |  |  |  |  |  |  |  |
| S.E. of regression | 6486.587 | Akaike info criterion | | 20.58958 |  |  |  |  |  |  |  |
| Sum squared resid | 7.15E+08 | Schwarz criterion | | 20.83754 |  |  |  |  |  |  |  |
| Log likelihood | -221.4853 | Hannan-Quinn criter. | | 20.64799 |  |  |  |  |  |  |  |
| Durbin-Watson stat | 1.604378 |  |  |  |  |  |  |  |  |  |  |

**Table J: Mean costs per SVR and 95% CI in the US output**

| Dependent Variable: US_COST_PER_SVR2 | | | |  |  |  | Coefficient Confidence Intervals | | |  |  |
| --- | --- | --- | --- | --- | --- | --- | --- | --- | --- | --- | --- |
| Method: Least Squares | |  |  |  |  |  | Date: 04/30/16 Time: 08:10 | |  |  |  |
| Date: 04/30/16 Time: 08:09 | |  |  |  |  |  | Sample: 1 22 |  |  |  |  |
| Sample: 1 22 |  |  |  |  |  |  | Included observations: 22 | |  |  |  |
| Included observations: 22 | |  |  |  |  |  |  |  |  |  |  |
|  |  |  |  |  |  |  |  |  |  | 95% CI |  |
| Variable | Coefficient | Std. Error | t-Statistic | Prob. |  |  | Variable | Coefficient |  | Low | High |
|  |  |  |  |  |  |  |  |  |  |  |  |
| STEP1 | 51350.09 | 4713.417 | 10.89445 | 0 |  |  | STEP1 | 51350.09 |  | 41405.65 | 61294.53 |
| STEP2 | 72770.76 | 4713.417 | 15.43907 | 0 |  |  | STEP2 | 72770.76 |  | 62826.32 | 82715.2 |
| STEP3 | 76392.66 | 2721.293 | 28.0722 | 0 |  |  | STEP3 | 76392.66 |  | 70651.23 | 82134.09 |
| STEP4 | 101134.4 | 2721.293 | 37.16411 | 0 |  |  | STEP4 | 101134.4 |  | 95392.99 | 106875.8 |
| STEP5 | 93392.91 | 2721.293 | 34.31932 | 0 |  |  | STEP5 | 93392.91 |  | 87651.48 | 99134.33 |
|  |  |  |  |  |  |  |  |  |  |  |  |
| R-squared | 0.868578 | Mean dependent var | | 85170.98 |  |  |  |  |  |  |  |
| Adjusted R-squared | 0.837655 | S.D. dependent var | | 16543.64 |  |  |  |  |  |  |  |
| S.E. of regression | 6665.779 | Akaike info criterion | | 20.64408 |  |  |  |  |  |  |  |
| Sum squared resid | 7.55E+08 | Schwarz criterion | | 20.89204 |  |  |  |  |  |  |  |
| Log likelihood | -222.0849 | Hannan-Quinn criter. | | 20.70249 |  |  |  |  |  |  |  |
| Durbin-Watson stat | 1.517962 |  |  |  |  |  |  |  |  |  |  |
